# The Use of the “Respiratory Rate Oxygenation (ROX) Index” in the Assessment of Respiratory Support and Observation of Its Outcome in COVID-19 Patients With Acute Respiratory Failure

**DOI:** 10.7759/cureus.39529

**Published:** 2023-05-26

**Authors:** Abdullah T Bhuiyan, Mosharaf Hossain, Miftahul J Khan, Bablu Hossain, Shamim A Sultana, Farzana Sharmin, AKM Akhtaruzzaman

**Affiliations:** 1 Department of Anaesthesia, Analgesia and Intensive Care Medicine, Dhaka Medical College Hospital, Dhaka, BGD; 2 Department of Anaesthesia, National Institute of Cancer Research and Hospital, Dhaka, BGD; 3 Department of Anaesthesia, Sir Salimullah Medical College Mitford Hospital, Dhaka, BGD; 4 Department of Anaesthesia, Rangpur Medical College Hospital, Rangpur, BGD; 5 Department of Anaesthesia, Analgesia and Intensive Care Medicine, Bangabandhu Sheikh Mujib Medical University, Dhaka, BGD; 6 Department of Anaesthesiology, National institute of Traumatology and Orthopaedic Rehabilitation, Dhaka, BGD

**Keywords:** continuous positive airway pressure, high flow nasal cannula, respiratory support, acute respiratory failure, rox index, covid-19

## Abstract

Background: COVID-19 infection caused by the SARS-Cov-2 virus may result in severe acute respiratory failure and require respiratory support in the ICU.

Objective: The present study was designed to evaluate the role of the respiratory rate oxygenation (ROX) index in the assessment of the adequacy of non-invasive respiratory support the COVID-19 patients with acute respiratory failure and observe its outcome.

Materials and methods: This cross-sectional, observational study was conducted between October 2020 and September 2021 in the Department of Anaesthesia, Analgesia, and Intensive Care Medicine of Bangabandhu Sheikh Mujib Medical University (BSMMU), Dhaka, Bangladesh. A total of 44 patients with a confirmed diagnosis of COVID-19 with acute respiratory failure were enrolled in this study based on inclusion and exclusion criteria. Informed written consent was taken from the patient/patient’s guardian. Each patient underwent detailed history taking through physical examination and relevant investigations. All necessary information were recorded in a separate case record form. All the patients receiving high-flow nasal cannula (HFNC) were assessed at two hours, six hours, and 12 hours for variables of the ROX Index. The team of respective physicians was applied responsibly for determining HFNC failure to discontinue or deescalate respiratory support as a part of continuous positive airway pressure (CPAP) ventilation success. Each selected patient was observed for the duration of different types of respiratory support. CPAP failure or success, progression to mechanical ventilation, and data were collected from individual medical records. The patients who were successfully weaned from CPAP were recorded. The diagnostic accuracy of the ROX index was determined.

Results: The mean age of the patients was 65±8.80 years with a majority in the age group 61-70 years (36.4%). A male predominance was observed with 79.5% male and 20.5% female. Of all, HFNC failure was observed in 29.5% of patients. Oxygen saturation (SpO2), respiratory rate (RR), and ROX index were statistically worse at the sixth and 12th hour of initiation of HFNC (P<0.05). At a cut-off value of 3.90, the ROC curve showed 90.3% sensitivity and 76.9% specificity in predicting HFNC success (the area under the curve (AUC) was 0.909). Similarly, 46.2% of patients had CPAP failure. SpO2, RR, and ROX index were found statistically worse among those patients at the sixth and 12th hour of CPAP therapy (P<0.05). The ROC curve showed 85.7% sensitivity and 83.3% specificity at a cut-off value of 2.64 in predicting CPAP success (the AUC was 0.881).

Conclusion: The ROX index's clinical score form, which does not require lab findings or sophisticated computation techniques, is its key benefit. The study findings recommend the use of the ROX index to predict the outcome of respiratory support in acute respiratory failure in COVID-19 patients.

## Introduction

The respiratory rate oxygenation (ROX) index is an early predictor of failure of high-flow nasal cannula (HFNC) and continuous positive airway pressure (CPAP) ventilation [1]. The ROX index was recently introduced as an early predictor of intubation and mechanical ventilation in patients with acute hypoxic respiratory failure treated with HFNC/CPAP. The ROX index was defined as the ratio of oxygen saturation (SpO2)/fraction of inspired oxygen (FiO2), which has a positive association with HFNC/CPAP success, to respiratory rate (RR), which has an inverse association [1]. It is easily derived from commonly recorded variables measured in a non-invasive manner. However, it remains to be seen if the ROX index will perform as well in patients with respiratory failure from other causes than pneumonia [2]. The utility of the ROX index, defined as the ratio of SpO2 as measured by pulse oximetry/FiO2 to RR, for determining which patients treated with HFNC will not require intubation [3]. Therefore, the ROX index has been proposed as a tool to predict the adequacy of HFNC and CPAP as well as outcome in COVID-19 patients with acute respiratory failure who are admitted into the ICU [4-6].

The COVID-19 pandemic was caused by SARS-Cov-2. Most of the patients who require ICU support due to COVID-19 infection showed severe acute respiratory failure and needed respiratory support [4-7]. Initially, early intubation and mechanical ventilation were thought to be the cornerstone of treatment, but complications of endotracheal intubation and mechanical ventilation made it unpopular [5. Non-invasive ventilation was provided via nasal prong, HFNC, and CPAP ventilation [4-6]. Delaying endotracheal intubation and mechanical ventilation as indicated in those patients with respiratory distress increases ICU morbidity, complication, and mortality [8]. In COVID-19 respiratory failure, the ROX index is an easily available and adaptable tool to predict the application of different types of respiratory support [4-6]. The use of the ROX index might be beneficial for COVID-19 respiratory failure patients and also helpful to a physician to take the decision of early intubation and other interventions, thus, reducing ICU mortality and length of ICU stay, identification of patients at high risk of in-hospital death, and relocation of resources and reducing the cost and improving the future quality of ICU service [7-9]. The utility of the ROX index has been studied in different countries; however, no such report has been available in our country to date. Hence, we proposed the present study to use the "ROX index" in the assessment of respiratory support and observe its outcome in COVID-19 patients with acute respiratory failure [10].

## Materials and methods

This cross-sectional, observational study was conducted in the Department of Anaesthesia, Analgesia, and Intensive Care Medicine of Bangabandhu Sheikh Mujib Medical University (BSMMU), Dhaka, Bangladesh. A total of 44 patients with a confirmed diagnosis of COVID-19 with acute respiratory failure were enrolled in this study based on inclusion and exclusion criteria.

Inclusion criteria were COVID-19 patients diagnosed using RT-PCR with a history of acute respiratory failure who fulfill the following criteria: (1) patients having hypoxic respiratory failure, (2) aged more than 18 years, (3) receiving high-flow nasal oxygen support. Exclusion criteria were (1) patients with chronic obstructive pulmonary disease, (2) any requirement of emergency intubation for CPR, (3) severe hemodynamic instability, (4) patients with any signs of encephalopathy, (5) respiratory failure caused by neurologic disease or status asthmaticus, and (5) more than two organ failures.

Informed written consent was taken from the patient/patient’s guardian. Each patient underwent detailed history taking through physical examination and relevant investigations. All necessary information were recorded in a separate case record form. All the patients receiving HFNC were assessed at two hours, six hours, and 12 hours for variables of the ROX index. In patients receiving HFNC oxygen therapy, the ROX index defined as the ratio of SpO2 as determined by pulse oximetry/FIO2 to RR was evaluated as a predictor of the need for intubation. The team of respective physicians was applied responsibly for determining HFNC failure to discontinue or deescalate respiratory support as a part of CPAP ventilation success. Each selected patient was observed for the duration of different types of respiratory support. CPAP failure or success, progression to mechanical ventilation, and data were collected from individual medical records. The patients who were successfully weaned from CPAP were recorded.

The results were presented in tables. Quantitative data were expressed as mean and standard deviation, while qualitative data were expressed as frequency and percentage. The association between variables was determined by using an unpaired Student's t-test. Moreover, receiver operating characteristic (ROC) curves were plotted to assess the performance of the ROX index predicting the success of HFNC and CPAP. The power of prediction was quantified by the area under the curve (AUC) with 95% confidence intervals, as the larger the AUC is, the better the predictive accuracy is. A p-value of <0.05 was considered statistically significant. All the statistical analyses were performed using SPSS Statistics version 23.0 (IBM Corp. Released 2015. IBM SPSS Statistics for Windows, Version 23.0. Armonk, NY: IBM Corp.). Ethical approval was obtained from the Institutional Review Board of Bangabandhu Sheikh Mujib Medical University (BSMMU), Dhaka, Bangladesh (Memo: BSMMU/2021/568, Dated: 21.01.2021).

## Results

The mean age of the patients was 65.20±8.80 years. Among them, 79.5% were male and 20.5% were female. The mean duration of COVID-19 symptoms was 11.82±5.60 days, and the mean duration since COVID-19 detection was 6.59±4.01days (Table 1).

**Table 1:** Demographic characteristics of the patients (n=44)

Variables	Mean±SD
Age (years)	65.20±8.80
Sex (male-female ratio)	4:1
Duration of COVID-19 symptoms (days)	11.82±5.60
Duration since COVID-19 detection (days)	6.59±4.01

**Figure 1:**
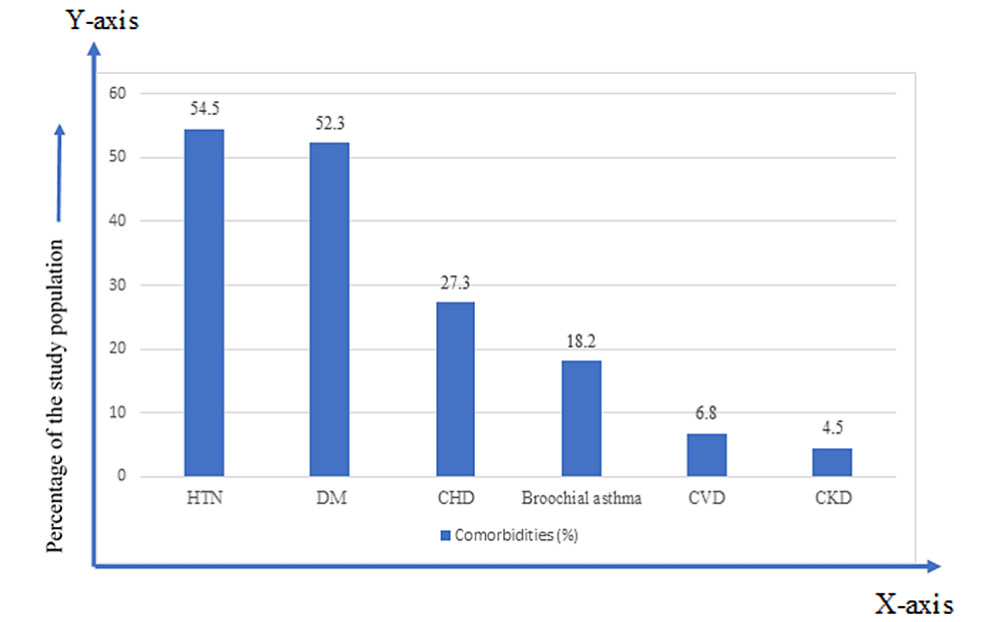
The presence of comorbidities among the admitted patients (n=44)

About 22.7% of the patients had no comorbidity. The most common comorbidities were hypertension (54.5%), diabetes mellitus (52.3%), chronic heart disease (27.3%), bronchial asthma (18.2%), cerebrovascular disease (6.8%), and chronic kidney disease (4.5%) (Figure 1).

Presenting clinical features among the patients include fever (81.8%), cough (72.3%), body ache 43.2%), breathlessness (31.8%), chest pain (43.2%), nausea and/or vomiting (20.6%), diarrhea (20.6%), altered smell (20.6%), fatigue (20.5%), headache (15.9%), sore throat (15.9%), and runny nose (9.5%) (Table 2).

**Table 2:** Clinical features of the admitted patients (n=44)

Clinical presentation	n	%
Fever	36	81.8
Cough	32	72.3
Body ache	19	43.2
Chest pain	19	43.2
Breathlessness	14	31.8
Fatigue	9	20.5
Nausea and/or vomiting	9	20.6
Diarrhea	9	20.6
Altered smell	9	20.6
Headache	7	15.9
Sore throat	7	15.9
Runny nose	4	9.5

**Figure 2:**
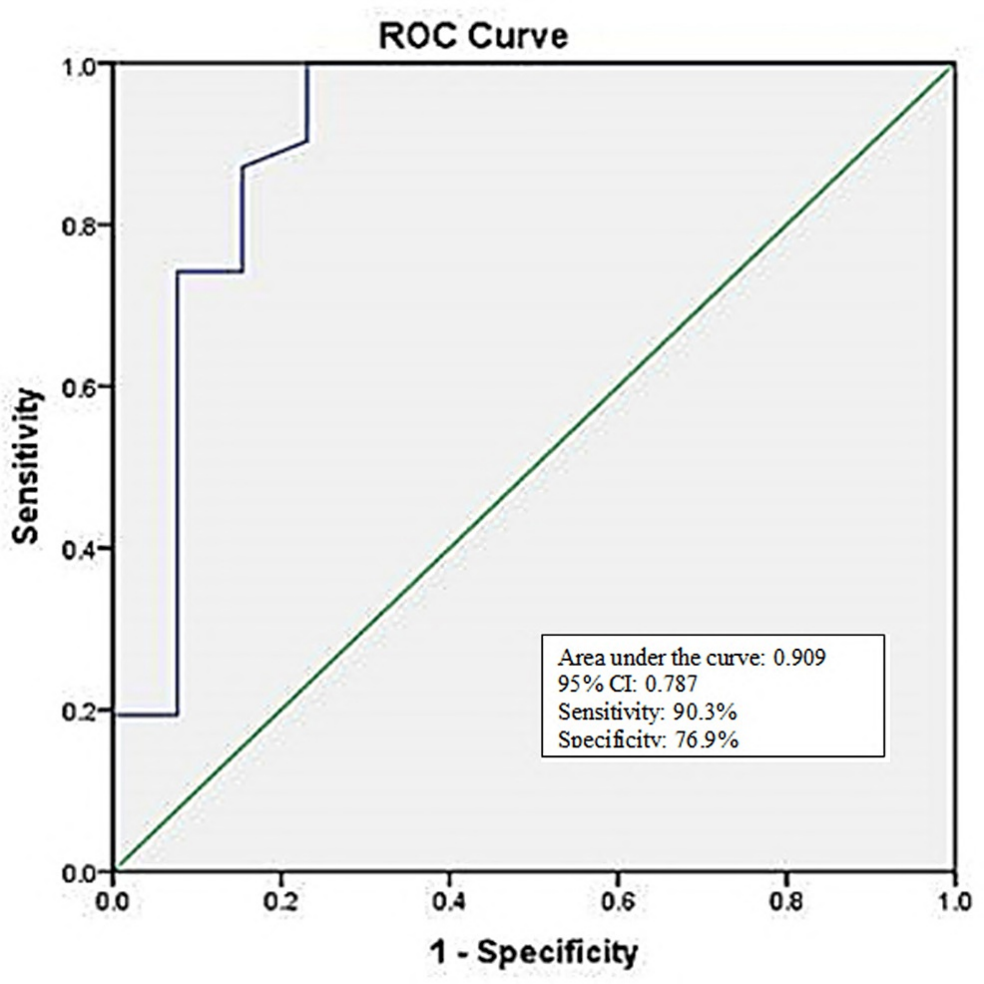
ROC of the "ROX index" at the 12th hour of HFNC therapy in the prediction of HFNC success (n=44) ROC: receiver operating characteristic

At a cut-off value of ≤3.90, the "ROX index" at the 12th hour of HFNC could predict successful outcomes in 28 patients (out of 31). Among the rest 13 patients with HFNC failure, we could predict 10 patients. The ROC curve showed 90.3% sensitivity and 76.9% specificity; the AUC was 0.909 (Tables 3-4 and Figure 2).

**Table 3:** ROX index and its components between HFNC success and failure (n=44) HFNC: high-flow nasal cannula, SpO2: oxygen saturation, RR: respiratory rate, ROX index: respiratory rate oxygenation index

Variables	HFNC success	HFNC failure	p-value
SpO2			
2nd hour	85.54±5.62	80.84±14.66	>0.05^NS^
6th hour	85.61±5.09	79.61±7.59	<0.05^S^
12th hour	87.61±4.89	77.30±10.90	<0.001^S^
RR			
2nd hour	24.61±3.79	25.00±2.48	>0.05^NS^
6th hour	23.45±3.90	26.99±3.17	<0.001^S^
12th hour	20.01±1.39	25.32±4.24	<0.05^S^
ROX index			
2nd hour	3.49±0.732	3.23±0.57	>0.05^NS^
6th hour	3.72±0.56	3.17±0.13	<0.05^S^
12th hour	4.39±0.34	2.90±0.45	<0.001^S^

**Table 4:** Diagnostic accuracy of the ROX index at the 12th hour of HFNC in the prediction of HFNC success (n=44) HFNC: high-flow nasal cannula

	HFNC success		
	Success	Failure	Total
>3.9	28 (TP)	3 (FP)	31 (TF+FP)
≤3.9	3 (FN)	10 (TN)	13 (FN+TN)
	31 (TP+FN)	13 (FP+TN)	44

**Figure 3:**
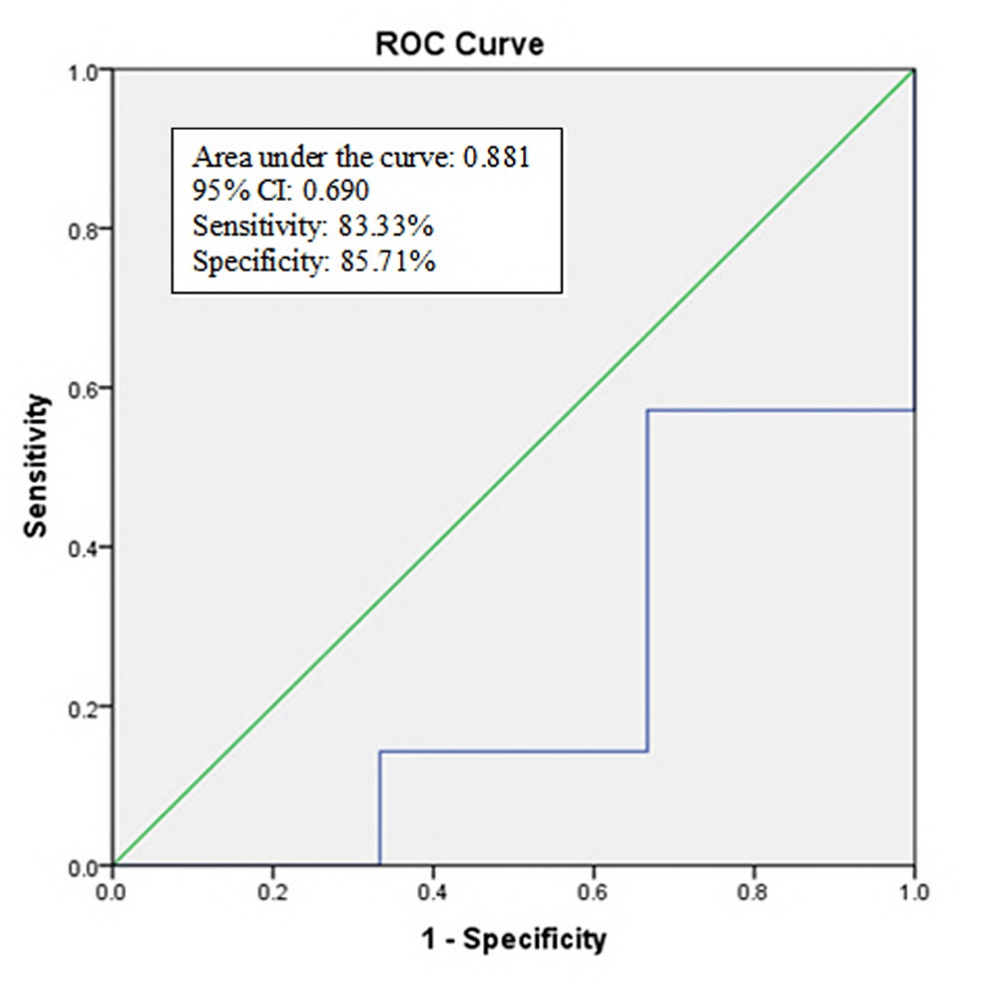
ROC of the "ROX index" at the 12th hour of CPAP for the prediction of CPAP success (n=13) ROC: receiver operating characteristic

At a cut-off value of ≤2.64, the "ROX index" at the 12th hour of CPAP could predict successful outcomes in five patients (out of six). Among the rest seven patients with CPAP failure, we could predict six patients. The ROC curve showed 83.33% sensitivity and 85.71% specificity; the AUC was 0.881 (Tables 5-6 and Figure 3).

**Table 5:** ROX index and its components between CPAP success and failure (n=13) CPAP: continuous positive airway pressure, SpO2: oxygen saturation, RR: respiratory rate, ROX index: respiratory rate oxygenation index

Variables	CPAP success	CPAP failure	p-value
SpO2			
2nd hour	75.83 ± 9.19	69.14 ± 14.06	>0.05^NS^
6th hour	85.28 ± 4.25	66.66 ± 5.16	<0.001^S^
12th hour	89.42 ± 1.98	62.32 ± 2.28	<0.001^S^
RR			
2nd hour	25.85 ± 2.11	24.16 ± 2.31	>0.05^NS^
6th hour	24.71 ± 1.66	29.32 ± 1.32	>0.05^NS^
12th hour	23.71 ± 1.39	29.88±1.11	<0.001^S^
ROX index			
2nd hour	3.16 ± 0.52	2.65 ± 0.15	>0.05^NS^
6th hour	3.08 ± 0.29	2.3± 0.25	<0.05^S^
12th hour	3.77 ± 0.218	2.12 ± 0.09	<0.001^S^

**Table 6:** Diagnostic accuracy of the ROX index at the 12th hour of CPAP for the prediction of CPAP success (n=13) CPAP: continuous positive airway pressure

	CPAP success		
	Success	Failure	Total
>2.64	5 (TP)	1 (FP)	6 (TF+FP)
≤2.64	1 (FN)	6 (TN)	7 (FN+TN)
	6 (TP+FN)	7 (FP+TN)	13

## Discussion

COVID-19 has a wide spectrum of severity as mild (81%), severe (14%), and critical (5%) [10]. A high number of infected patients develop pneumonia, and many of them get complicated with acute respiratory distress syndrome and eventually require respiratory support [11].

Determining the disease progression by subtyping and predicting the outcome of COVID-19 patients might aid in targeting treatment and medical resource allocation according to patients’ risk [8,9]. The patients who need respiratory support undergo continuous crucial follow-up because a failure of respiratory support is an indication of invasive mechanical ventilation and ICU support [5-7]. However, continuous monitoring of those patients through arterial blood gas analysis along with other investigations is expensive for our resource-poor settings. Therefore, this study was designed to evaluate the role of the ROX index as a predictor of the outcome of non-invasive respiratory support of COVID-19 patients.

In our study, the mean age of the patients was 65±8.80 years with a majority in the age group 61-70 years (36.4%). A similar age distribution was observed in the studies by Perrotta et al. [12] and Tehrani et al. [13]. In the present study, male predominance was observed with 79.5% male and 20.5% female patients. The male predominance has been consistent with previous studies done by Grasselli et al. [14] and Forsblom et al. [15]. It has been hypothesized that the X chromosome contains a high density of immune-related genes and regulatory elements that are extensively involved in both innate and adaptive immunity [16]. The commonest comorbidity in study patients was hypertension and diabetes mellitus which were present in 54.5% and 52.3% of the patients, respectively. Li et al. [17] also found hypertension as the most common comorbidity in COVID-19 patients in a meta-analysis. The clinical features among our patients include fever, cough, body ache, breathlessness, chest pain, fatigue, headache, sore throat, nausea and/or vomiting, diarrhea, altered smell, and runny nose. Nonetheless, this study included only those admitted patients who were RT-PCR positive which explained the reason behind the findings. However, clinical features may vary in different regions, ethnicities, and hospital setups [18].

HFNC success was observed in more than two-thirds of patients. SpO2, RR, and ROX index were statistically lower among patients with HFNC failure at the sixth and 12th hour of HFNC therapy. At a cut-off value of 3.9, the ROX index showed 90.32% sensitivity, 76.92% specificity, 90.32% positive predictive value (PPV), 76.92% negative predictive value (NPV), and 86.36% accuracy. About 13 patients were receiving CPAP, and among them, 46.2% were observed with CPAP failure. SpO2, RR, and ROX index all were statistically worse among patients with CPAP failure at the sixth and 12th hour of CPAP therapy. At a cut-off value of 2.64, 83.33% sensitivity, 85.71% specificity, 83.33% PPV, 85.71% NPV, and 84.61% accuracy. Mellado-Artigas et al. [18] did a similar type of study and observed at a ROX index of >4.4, ROX was associated with the requirement intubation. Patel et al. [19] and Hu et al. [20] also observed similar findings in their studies.

Although optimum care was taken by the researchers in every step of this study, some limitations still exist. The main limitation of our study was that it was done on a small sample in a single center due to time constraints and a limited budget. Therefore, it would be practically challenging to generalize the finding and apply them to the specified population. Moreover, its design was cross-sectional, which limits its capability to draw a causal inference, which could be done in a prospective cohort study. However, with our results, it is still convincible that the study was an appropriate one because of its ready availability for clinical use, cost-effectiveness, and accessibility. However, a large-scale study with multi-center involvement is recommended to allow the findings of the study to be generalized to our reference population.

## Conclusions

The ROX index clinical score form, which does not require lab findings or sophisticated computation techniques, is its key benefit. Our data suggest that the ROX index had 90% sensitivity and 76% specificity in predicting HFNC success and 83% sensitivity and 87% specificity in predicting CPAP success in patients with COVID-19 respiratory failure. Hence, the ROX index could be a useful predictor of failure of HFNC and CPAP in COVID-19-related acute respiratory insufficiency. The ROX index is non-invasive, fast, easily reproducible, and tremendously inexpensive. It is a rapid and simple tool that can be done at the bedside of the patients.
